# An evaluation of the effects and safety of Zuogui pill for treating osteoporosis: Current evidence for an ancient Chinese herbal formula

**DOI:** 10.1002/ptr.6908

**Published:** 2020-10-21

**Authors:** Jinyu Li, Kai Sun, Baoyu Qi, Guiyu Feng, Wei Wang, Qi Sun, Chengying Zheng, Xu Wei, Yusong Jia

**Affiliations:** ^1^ Department of Orthopaedics Dongzhimen Hospital, Beijing University of Chinese Medicine Beijing China; ^2^ Department of Spine Wangjing Hospital, China Academy of Chinese Medical Sciences Beijing China; ^3^ Department of Graduate School Beijing University of Chinese Medicine Beijing China; ^4^ Department of Scientific Research Wangjing Hospital, China Academy of Chinese Medical Sciences Beijing China

**Keywords:** Chinese herbal formula, osteoporosis, systematic review, Zuogui pill

## Abstract

The aim of this study is to systematically evaluate existing evidence of the Chinese herbal formula, Zuogui pill (ZGP), for the treatment of osteoporosis. A systematic literature search was performed in six electronic databases. The authors independently extracted data in pairs and evaluated the risk of bias. A total of 221 articles were identified initially, of which 12 relevant studies were enrolled. The primary outcome was fracture incidence and bone mineral density (BMD) at different sites. Bone metabolism markers, clinical symptoms, quality of life, and adverse events or adverse drug reactions (ADRs) were secondary outcomes. The results showed that ZGP, combined with anti‐osteoporosis drugs, significantly increased BMD at the lumbar spine, Ward's area, and total hip. In terms of markers for improved bone metabolism, ZGP plus conventional drugs dramatically improved the levels of alkaline phosphatase, bone Gla protein, bone alkaline phosphatase, and tartrate‐resistant acid phosphatase. Gastrointestinal discomfort, dizziness, and fatigue were found in the combined therapy group. Although the results indicate that ZGP is a potential candidate for osteoporosis, evidence remains insufficient. Further rigorously designed and high‐quality trials with a larger sample size are warranted to verify the current conclusions.

## 1 INTRODUCTION

Osteoporosis is a metabolic bone disease of reduced bone strength that predisposes older individuals to an elevated risk for fracture (Siris et al., 2014). In China, the prevalence of osteoporosis ranged from 14.94% before 2008 to 27.96% during the period spanning 2012–2015, and the rate was higher in females than in males (25.41% vs. 15.33%) (Chen, Li, & Hu, 2016). Similarly, 29.9% of women and 16% of men older than 50 years have osteoporosis, based on the National Bone Health Alliance diagnostic criteria in the United States (Wright, Saag, Dawson‐Hughes, Khosla, & Siris, 2017). In addition, a population‐based cohort study indicated that the majority of fractures occur within the osteoporosis threshold; thus, the management of patients with osteopenia or osteoporosis should be emphasized (Trajanoska et al., 2018).

To date, calcium and/or vitamin D supplementation are the standard choices for osteoporosis treatment (Aspray et al., 2014; Reymondier et al., 2013). Drug treatment, including bisphosphonates or denosumab, is also recommended to reduce the risk of vertebral or hip fractures in patients with osteoporosis. For postmenopausal women, oestrogen therapy, menopausal oestrogen plus progestogen therapy, or raloxifene is appropriate (Qaseem, Forciea, McLean, & Denberg, 2017). Nevertheless, despite the availability of multiple anti‐osteoporosis agents with distinct pharmacological functions and single‐pill combination pharmacotherapy, the target treatment effect is not achieved in large numbers of osteoporotic patients, and the prevention of osteoporotic fracture remains suboptimal (Burch et al., 2014; Ishtiaq, Fogelman, & Hampson, 2015). Accordingly, there is a clear requirement for newer therapeutic options or agents. In recent years, the increasing use of complementary and alternative medicine, including Chinese herbal medicine, for treating osteoporosis has attracted widespread attention (Li et al., 2017; Wang et al., 2018; Zhang et al., 2016).

Zuogui pill (ZGP) is a commonly used Chinese herbal formula invented by *Zhang Jingyue*, as outlined in *Jingyue Quanshu* (Jingyue's Complete Works) in 1624. ZGP is composed of eight Chinese herbs, including Shudi (Radix Rehmanniae Preparata), Shanyao (Rhizoma Dioscoreae), Gouqizi (Fructus Lycii), Shanyurou (Fructus Corni), Niuxi (Radix Achyranthis Bidentatae), Tusizi (Semen Cuscutae), Guibanjiao (Colla Plasti Testutinis), and Lujiaojiao (Colla Cornus Cervi). All of these herbs have been documented in the *Pharmacopoeia of the People's Republic of China* (2015 edition). According to the theory of traditional Chinese medicine (TCM), ZGP is more adaptable to kidney‐yin deficiency syndrome (Liu, Cai, & Chen, 1997), which manifests as dysphoria with feverish sensation in the chest, palms and soles, hot flashes, night‐time sweating, sore waist and knees, and dry mouth (Lian et al., 2014). Currently, the formula is often prescribed by clinicians in China for the management of osteoporosis (Liu et al., 2011). More importantly, ZGP is also the drug recommended in the latest osteoporosis clinical diagnosis and treatment guidelines issued by authoritative academic institutions in China, such as the Chinese Medical Association of Osteoporosis and Bone Mineral Research (Chinese Medical Association of Osteoporosis and Bone Mineral Research, 2017), the Chinese Association of Chinese Medicine (Chinese Association of Chinese Medicine, 2020), and the Chinese Association of Integrative Medicine (Chinese Association of Integrative Medicine, 2019). With further research, the mechanism of action of ZGP is beginning to be elucidated. One basic study found that ZGP could alleviate glucocorticoid‐induced osteoporosis by up‐regulating the expression of the pro‐oncogene Wnt‐1, low‐density lipoprotein receptor‐related protein 5, and beta‐catenin (Liu et al., 2011). Another recent experimental study demonstrated that the formula, ZGP, might improve dexamethasone‐induced osteoporosis by inhibiting phosphorylation of transforming growth factor‐beta and mothers against decapentaplegic homologue 3, as revealed using zebrafish larvae (Yin et al., 2018). Although a number of relevant clinical trials on the effect and safety of ZGP have been conducted, related systematic reviews or meta‐analyses of randomized controlled clinical trials, reporting ZGP for osteoporosis, are unavailable to date. In this study, we evaluated the effect and safety of ZGP among patients with osteoporosis to provide evidence for clinical practice and scientific research.

## 2 METHODS

### 2.1 Search strategy and selection criteria

A systematic search of PubMed, Excerpta Medica Database (EMBASE), Cochrane Central Register of Controlled Trials (CENTRAL), and three Chinese electronic databases, including National Knowledge Infrastructure (CNKI), Wanfang Digital Periodicals (WANFANG), Chinese Science and Technology Periodicals (VIP) database, was conducted to identify randomized controlled trials (RCTs) of ZGP for patients with osteoporosis. The search strategy was established using the Participant, Intervention, Comparator, Outcome (PICO) framework, as suggested in the Preferred Reporting Items for Systematic Reviews and Meta‐Analyses (PRISMA) protocol guidelines (Shamseer et al., 2015). The strategy was piloted in November 2019, and all relevant literature was searched from inception to January 31, 2020. The following search terms were used in separate or combined ways: “osteoporosis”; “primary osteoporosis”; “postmenopausal osteoporosis”; “senile osteoporosis”; “age‐related osteoporosis”; “bone loss”; “osteopenia”; “Zuogui Pill”; “Zuogui granules”; “randomized controlled trial”. No limits were applied with regard to language, publication year, sex, or race. The detailed search terms are shown in the Data S1 (supplementary material).

All included studies are required to comply with the “PICOS” principle, the details of which are as follows: (a) Participants (P): Patients diagnosed with primary or secondary osteoporosis (Kanis, Melton, Christiansen, Johnston, & Khaltaev, 1994; The Osteoporosis Committee of China Gerontological Society, 2000). Postmenopausal osteoporosis and senile osteoporosis are considered primary osteoporosis; (b) Intervention (I): ZGP or ZGP combined with anti‐osteoporosis drugs or alendronate; (c) Control (C): All types of conventional treatments recommended in clinical practice guidelines were included; (d) Outcomes (O): The outcomes include at least one of the following: fracture incidence, quality of life, clinical symptoms (such as pain, muscle fatigue, and limited mobility), death directly or indirectly attributed to osteoporosis, adverse effects, BMD, and biochemical markers of bone turnover (Liu, Liu, & Xia, 2014; Xu et al., 2017); (e) Study design (S): RCTs. The inclusion criteria of the included studies are summarized in Table 1.

**TABLE 1:** Inclusion criteria and exclusion criteria for the studies

Selection criteria	Details
Inclusion criteria	(a) Participants (P): Patients diagnosed with primary or secondary osteoporosis^a^;
	(b) Intervention (I): ZGP or ZGP combined with anti‐osteoporosis drugs or alendronate;
	(c) Control (C): Routine anti‐osteoporosis drugs group or a group exposed to different interventions recommended in clinical practice guidelines;
	(d) Outcomes (O): The outcomes include at least one of the following: Fracture incidence, quality of life, clinical symptoms (such as pain, muscle fatigue, and limited mobility), death directly or indirectly attributed to osteoporosis, adverse effects, BMD, and biochemical markers of bone turnover;
	(e) Study design (S): RCT.
Exclusion criteria	(a) Studies with patients who could not be diagnosed with osteoporosis;
	(b) Studies of modified ZGP for osteoporosis;
	(c) Studies in which the control group was not an accepted intervention;
	(d) Reviews, animal experiments, and meeting abstracts;
	(e) Studies with insufficient data or duplicate publications.

Abbreviations: RCT, Randomized controlled trial; ZGP, Zuogui pill.

^a^ The diagnosis for osteoporosis should be in accordance with international criteria, such as World Health Organization criteria, where bone mineral density (BMD), namely, *T* score, ≤ − 2.5, is defined as osteoporosis (Kanis et al., 1994). In addition, Chinese criteria (peak bone mass [*M* ± *SD*]<M‐2 SD confers an osteoporosis diagnosis) were included (The Osteoporosis Committee of China Gerontological Society, 2000).

As the focus of the review was on the treatment effect of the original formula, we used the following exclusion criteria (Table 1): (a) Studies with patients who could not be diagnosed with osteoporosis; (b) Studies of modified ZGP for osteoporosis; (c) Studies in which the control group was not an accepted intervention; (d) Reviews, animal experiments, and meeting abstracts; (e) Studies that presented insufficient data or were duplicate publications. The articles were reviewed and cross‐checked independently by three investigators (Jinyu Li, Kai Sun, and Baoyu Qi), and any disagreement was resolved by consensus among all three according to the above criteria.

### 2.2 Data extraction

The characteristics of the studies were independently extracted by two reviewers (Guiyu Feng and Wei Wang), including the first author, year of publication, sample size, type of osteoporosis, formation of ZGP, intervention of the experimental and comparison groups, all study outcomes, and duration of treatment.

Fracture incidence was the primary endpoint, and BMD at different sites, bone metabolism markers, clinical symptoms, quality of life, and adverse events or adverse drug reactions (ADRs) were secondary endpoints. Any disagreement was resolved by consensus.

### 2.3 Risk of bias and data synthesis of the included literature

Risk of bias and data extraction were completed by another two authors independently (Qi Sun and Chengying Zheng). The quality of the enrolled studies was evaluated using the Cochrane Handbook for Systematic Reviews of Interventions. Two reviewers (Qi Sun and Chengying Zheng) independently assessed the risk of bias in the included studies using the Modification of Cochrane Tool (RoB 2.0) (Sterne, Savović, Page, et al., 2019), with studies being classified as having a low, probably low, probably high, or high risk of bias. This evaluation was performed in the following domains: allocation sequence, allocation concealed, blinded, missing outcome data, selection of the reported results, and other problems. Any discrepancies were addressed through discussion, and if a consensus could not be reached, the opinion of an additional independent researcher (Yusong Jia) was sought.

RevMan 5.3 software provided by Cochrane Collaboration was applied to analyse the data. The results are presented as odds ratios (ORs) with 95% confidence intervals (CIs). For continuous outcomes, the weighted mean difference (WMD) was used when the units of outcomes were consistent. When *p* < .1 and *I* ^2^ > 50%, heterogeneity between studies was significant, considering the small sample sizes of the studies and heterogeneity in design (Higgins, Thompson, Deeks, & Altman, 2003). The random‐effect model was employed when heterogeneity between studies was confirmed; if there was no heterogeneity, the fixed‐effect model was applied to detect differences. Forest plots were generated for studies that measured the same outcome between groups. Funnel plots for publication bias were also drawn.

### 2.4 Quality of evidence

The certainty of evidence and strength of recommendations were assessed using the Grades of Recommendations Assessment, Development, and Evaluation (GRADE) method, which is considered a valid approach. GRADE mainly evaluates five aspects, including risk of bias, indirectness, imprecision, inconsistency, and publication bias. The large magnitude of an effect, plausible confounding, and a high dose–response gradient can upgrade the evidence level of the results (Atkins et al., 2004; Chen, Wang, Jiang, Kwong, & Gu, 2017). The evidence was evaluated as high, moderate, low, or very low quality according to the GRADE recommendations. GRADEPro software (available from gradepro.org) was used for the analysis. Two reviewers (Jinyu Li and Xu Wei) independently assessed the certainty of evidence and resolved any discrepancies by discussion.

## 3 RESULTS

### 3.1 Literature search results

**FIGURE 1:**
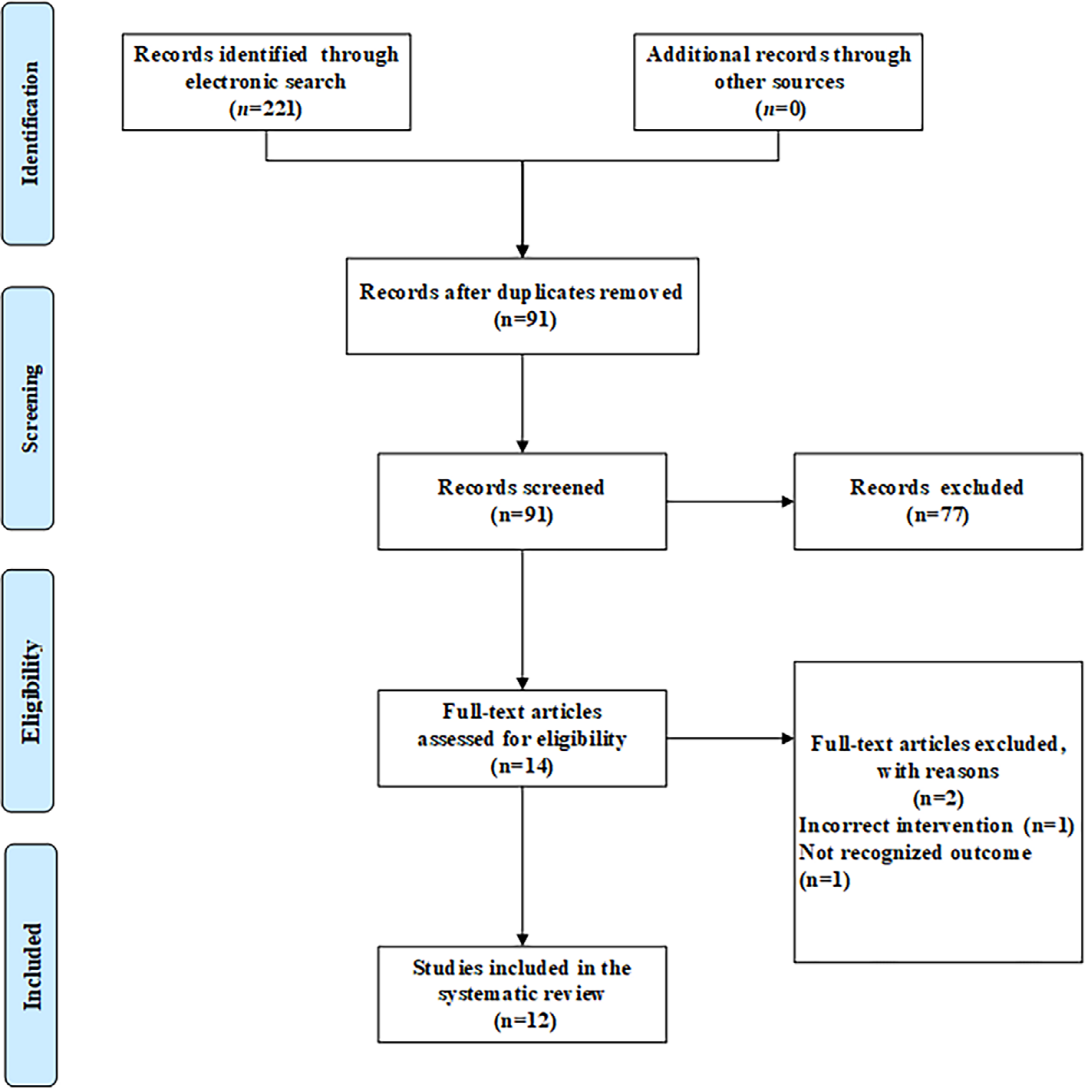
PRISMA 2009 flow diagram [Colour figure can be viewed at wileyonlinelibrary.com]

In total, 221 primary articles were initially identified in electronic databases, with 130 duplicates being excluded. Upon reviewing the titles and abstracts of the remaining records, 77 papers that were not relevant to the subject were excluded. Of the remaining 14 articles, two full texts that were animal experiments or involved other diagnoses were removed. Thus, 12 articles (Han, 2019; Li & Zhang, 2018; Li, Pan, & Cao, 2015; Liu et al., 2019; Ma, Jia, Cheng, & Gu, 2014; Ma, Liu, & Gao, 2018; Peng, 2010; Song, Li, & Ji, 2013; Wang et al., 2014; Wei, 2017; Yan, Lv, Li, & Zhu, 2012; Zhang, Zhang, Qiu, Lin, & Yang, 2018) including 13 RCTs met the inclusion criteria and were enrolled in the systematic review and meta‐analysis. All studies were found in the Chinese literature. Figure 1 presents the detailed flow diagram of the search and selection process.

### 3.2 General characteristics of the included articles

The included RCTs were published from 2010 to 2019, with a total of 1,104 subjects among various groups. The case numbers of the two RCTs included were small, and the total sample size was less than 60 cases (Li et al., 2015; Peng, 2010). Eleven articles examined the effect of ZGP on primary osteoporosis, whereas only one used the Chinese herbal formula to treat secondary osteoporosis (Wang et al., 2014). For the ZGP formulation, three studies used a decoction (Ma et al., 2014; Yan et al., 2012; Zhang et al., 2018), and the other used a pill form.

Two studies (Li et al., 2015; Peng, 2010) compared ZGP therapy alone versus conventional Western medicine alone based on a head‐to‐head study design. Two RCTs (Li & Zhang, 2018; Wang et al., 2014) compared ZGP with conventional medicine or placebo according to basic treatment. The other studies were add‐on study designs that compared ZGP plus conventional medicine and conventional medicine monotherapy. However, no “head‐to‐head design” placebo‐controlled trials were found.

One study reported fracture incidence (Song et al., 2013), one study reported pain symptoms (Zhang et al., 2018), one study reported quality of life (Li & Zhang, 2018), and six studies reported ADRs (Li et al., 2015; Ma et al., 2018; Peng, 2010; Wang et al., 2014; Wei, 2017; Zhang et al., 2018). In addition, all studies observed BMD at different sites, and only two (Li et al., 2015; Song et al., 2013) did not evaluate biochemical markers of bone turnover. All of the included studies involved at least 3 months of treatment. The general information of the included articles is provided in Table 2.

**TABLE 2:** General information of the articles included in this review

No	Reference	Number of subjects	Type of osteoporosis	Formation of ZGP	Experimental group	Comparison group	Study variables	Duration of treatment (months)
1	Peng (2010)	38	Primary osteoporosis	Pill	ZGP (9 g each time and three times a day)	Alfacalcidol (0.5–1 μg, once a day)	BMD Ca, P, UCa/Cr, ALP ADR	3
2	Yan et al. (2012)	120	Senile osteoporosis	Decoction	ZGP (a dose each day and twice a day) + comparison group	Caltrate tablets (0.6 g, once a day)	BMD UCa, ALP	3
3	Song et al. (2013)	75	Postmenopausal osteoporosis	Pill	ZGP (9 g each time and twice a day) + comparison group	Caltrate tablets (0.6 g, once a day)	Fracture incidence BMD (LS, FN, WA)	12
4	Ma et al. (2014)	100	Primary osteoporosis	Decoction	ZGP (100 mL each day and twice a day) + comparison group	Alendronate (70 mg, once a week) + Caltrate tablets (0.6 g, once a day)	BMD (LS) Ca, P, BGP, CT	6
5	Wang et al. (2014a)	60	Diabetes‐induced osteoporosis	Pill	ZGP (9 g each time and twice a day) + comparison group	Hypoglycaemic therapy	BMD (HB) Ca, P, ALP, PTH, BGP ADR	6
6	Wang et al. (2014)	60	Diabetes‐induced osteoporosis	Pill	ZGP (9 g each time and twice a day) + hypoglycaemic therapy	Caltrate tablets (0.6 g, once a day) + hypoglycaemic therapy	BMD (HB) Ca, P, PTH, ALP, BGP ADR	6
7	Li et al. (2015)	47	Primary osteoporosis	Pill	ZGP (9 g each time and twice a day)	Alendronate (10 mg, once a day)	BMD (LS, FN) ADR	6 (3 months in winter in the first and second year, respectively)
8	Wei (2017)	152	Postmenopausal osteoporosis	Pill	ZGP (9 g each time and twice a day) + comparison group	Alendronate (10 mg, once a day)	BMD (LS, FN, TH) ALP, BGP, 1,25‐(OH)_2_D_3_ ADR	3
9	Ma et al. (2018)	126	Senile osteoporosis	Pill	ZGP (9 g each time and twice a day) + comparison group	Salmon calcitonin (50 IU, once a day, intramuscular injection)	BMD (LS, WA, TH, HB) TNF‐α, BALP, TRACP, D‐Pyr/Cr ADR	3
10	Zhang et al. (2018)	84	Senile osteoporosis	Decoction	ZGP (a dose each day and twice a day + comparison group	Salmon calcitonin (50 IU, once a day, intramuscular injection) + Calcitriol (0.25 μg, twice a day) + Caltrate tablets (0.6 g, once a day) + Zoledronic acid (5 mg, once a year, intravenous drip)	VAS BMD (LS) 25(oh)_2_D ADR	3
11	Li et al. (2018)	60	Senile osteoporosis	Pill	ZGP (9 g each time and twice a day) + Caltrate tablets (0.6 g, once a day)	ZGP placebo (9 g each time and twice a day) + Caltrate tablets (0.6 g, once a day)	BMD (LS, FN, WA) P1NP, β‐CTX, β‐catenin SF‐36	6
12	Liu et al. (2019)	80	Postmenopausal osteoporosis	Pill	ZGP (9 g each time and three times a day) + comparison group	Alfacalcidol (0.5 μg, once a day) + Tibolone (2.5 mg, once a day)	BMD (LS, FN, WA) BGP, BALP, TRACP, E_2_, FSH, LH	6
13	Han (2019)	102	Postmenopausal osteoporosis	Pill	ZGP (9 g each time and twice a day) + comparison group	Alendronate (70 mg, once a week)	BMD (LS, FN) TRACP‐5b, CTSK	6

Abbreviations: ADR, adverse drug reaction; ALP, alkaline phosphatase; BALP, bone alkaline phosphatase; BGP, bone gla protein; Ca, blood calcium; CT, calcitonin; CTSK, cathepsin K; D‐Pyr/Cr, ratio of urine deoxypyridinoline and creatinine; E_2_, estradiol; FN, femoral neck; FSH, follicle‐stimulating hormone; HB, heel bone; LH, luteinizing hormone; LS, lumbar spine; P, blood phosphorus; P1NP, procollagen type 1 N‐terminal propeptide; SF‐36, short form 36 questionnaire; TH, total hip; TNF‐α, tumour necrosis factor α; TRACP, tartrated resistant acid phosphatase; UCa, urine calcium; UCa/Cr, ratio of urine calcium and creatinine; VAS, visual analogue scale; WA, wards area; ZGP, Zuogui pill; β‐CTX, β cross‐linked C‐telopeptide of type 1 collagen.

### 3.3 Risk of bias

Overall, only one study (Li & Zhang, 2018) was considered to have an unclear risk of bias; the other 11 studies presented a high risk of bias. The randomization scheme in six trials (Han, 2019; Li & Zhang, 2018; Ma et al., 2014, 2018; Wang et al., 2014; Zhang et al., 2018) using a random number table was considered reasonable. The domain of allocation concealment was uncertain in each article. Only one trial (Li & Zhang, 2018) conducted a placebo‐controlled design based on routine treatment. Seven trials had incomplete data (Li et al., 2015; Ma et al., 2018; Peng, 2010; Song et al., 2013; Wang et al., 2014; Wei, 2017; Zhang et al., 2018). For the domains blinding of outcome assessment and selective reporting, all studies were judged to be unclear because the detailed statistical plan and study protocol were not available. Other sources of bias related to the provision of baseline information for study participants were considered to be low. Table 3 shows the risk of bias for each study.

**TABLE 3:** Assessment of risk of bias for randomized controlled trials

Study ID	A	B	C	D	E	F
Peng (2010)	Probably low	Probably low	Probably high	Low	Probably high	Low
Yan et al. (2012)	Probably low	Probably low	Probably high	Probably low	Probably high	Low
Song et al. (2013)	Probably low	Probably low	Probably high	Low	Probably high	Low
Ma et al. (2014)	Low	Probably low	Probably high	Probably low	Probably high	Low
Wang et al. (2014)	Low	Probably low	Probably high	Low	Probably high	Low
Li et al. (2015)	Probably low	Probably low	Probably high	Low	Probably high	Low
Wei (2017)	Probably low	Probably low	Probably high	Low	Probably high	Low
Ma et al. (2018)	Low	Probably low	Probably high	Low	Probably high	Low
Zhang et al. (2018)	Low	Probably low	Probably high	Low	Probably high	Low
Li et al. (2018)	Low	Low	Low	Probably low	Probably high	Low
Liu et al. (2019)	Probably low	Probably low	Probably high	Probably low	Probably high	Low
Han (2019)	Low	Probably low	Probably high	Probably low	Probably high	Low

*Note*: “A” was the allocation sequence adequately generated?; “B” was the allocation adequately concealed?; “C” blinding was knowledge of the allocated interventions adequately prevented?; “D” was loss to follow‐up (missing outcome data) infrequent?; “E” are reports of the study free of selective outcome reporting?; “F” was the study apparently free of other problems that could put it at a risk of bias?; Low, low risk of bias; Probably low, Probably low risk of bias; Probably high, Probably high risk of bias.

### 3.4 Study results

According to the primary endpoint events and secondary endpoint measures, the results can be depicted as follows.

#### 3.4.1 Fracture incidence

One study (Song et al., 2013) found that the incidence for ZGP combined with Caltrate tablets (0/38) was lower than that for Caltrate tablets alone (2/37). The type of fracture in the control group was distal radius fracture and moderate vertebral compression fracture.

#### 3.4.2 BMD at all sites

**FIGURE 2:**
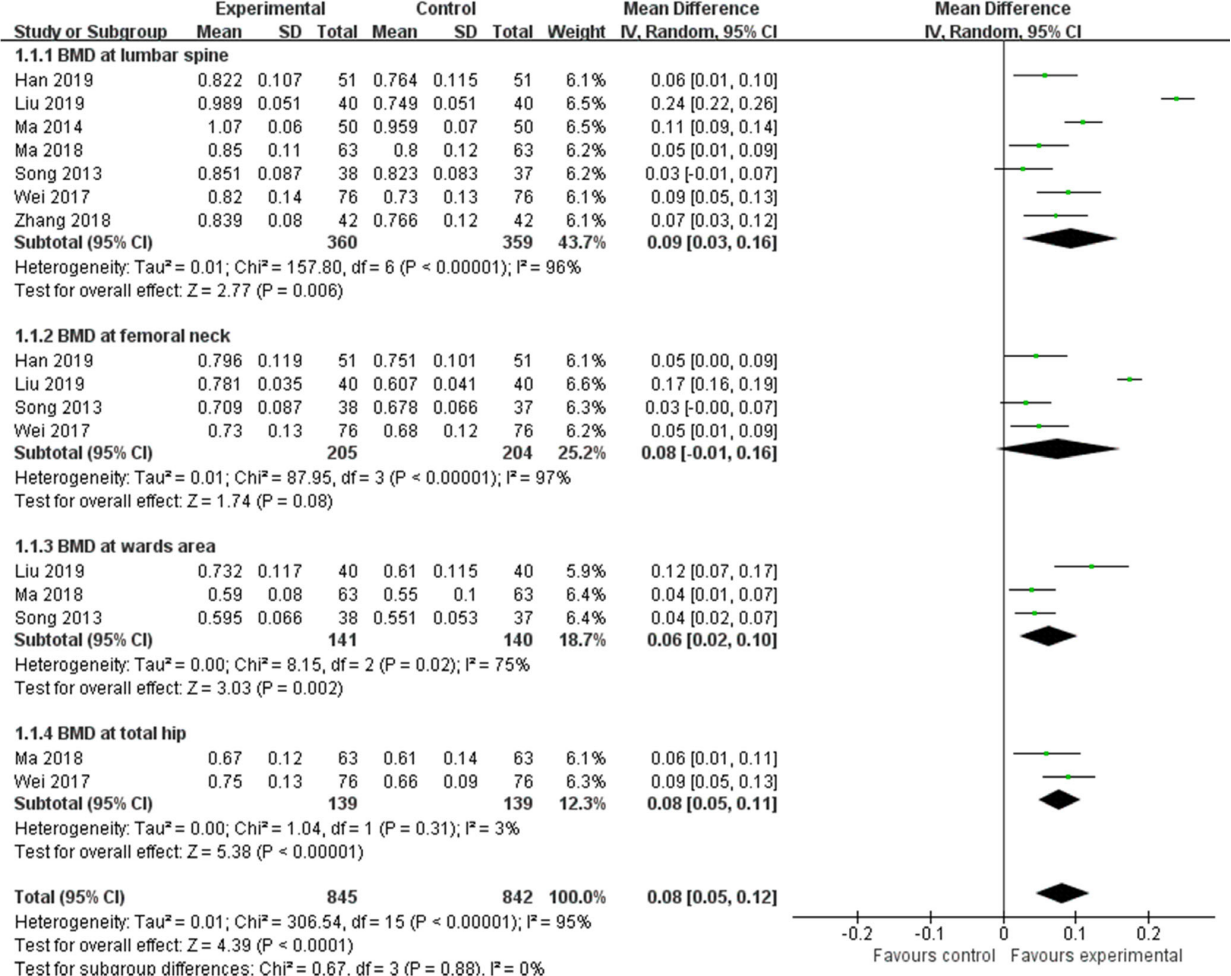
Forest plot of ZGP plus anti‐osteoporosis drugs compared with anti‐osteoporosis drugs alone for BMD at different anatomical sites [Colour figure can be viewed at wileyonlinelibrary.com]

One study (Peng, 2010) showed that BMD improvement in the ZGP group was significantly better than that in the alfacalcidol group (*p* < .01), but the sites of BMD were not reported clearly. Another study (Yan et al., 2012) showed that improvement of BMD with ZGP combined with Caltrate tablets was significantly better than for Caltrate tablets alone (*p* < .05), but the sites of BMD assessed were still uncertain. For the other studies, the analysis was presented based on BMD at different sites and interventions. Figure 2 illustrates the forest plot of ZGP plus anti‐osteoporosis drugs compared with anti‐osteoporosis drugs alone for BMD at different anatomical sites.

**FIGURE 3:**
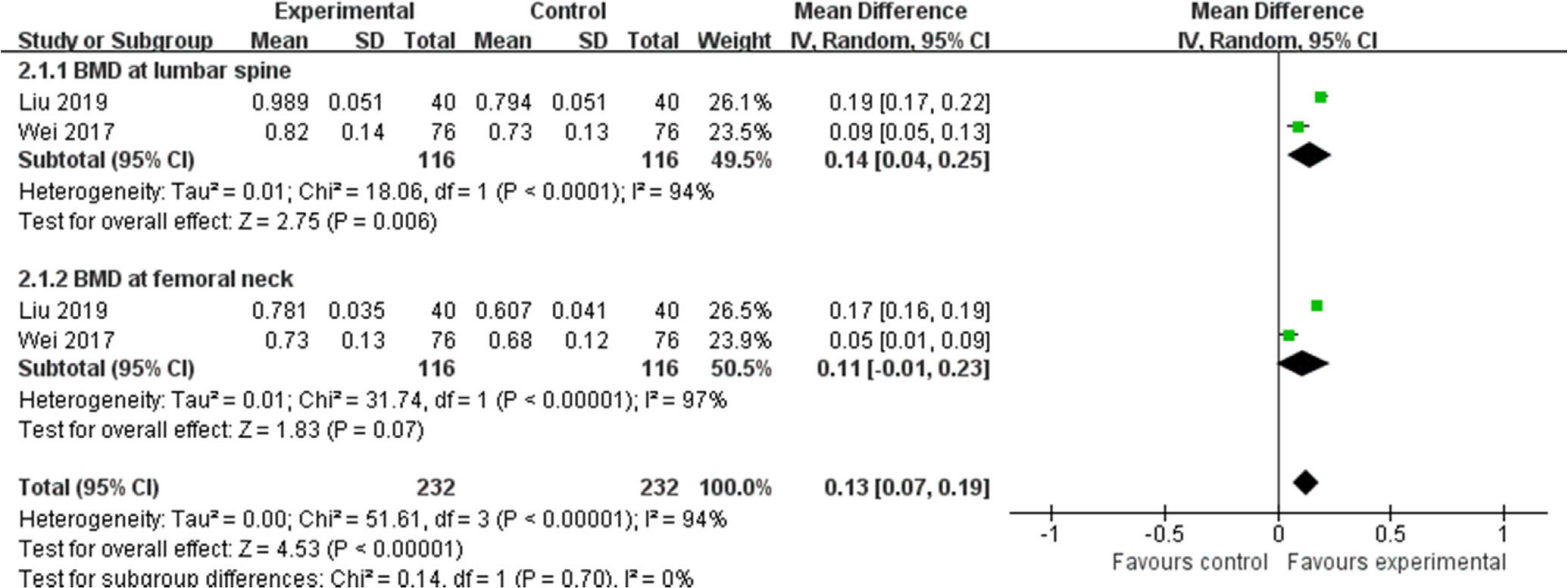
Forest plot of ZGP plus alendronate compared with alendronate alone regarding BMD at the lumbar spine and femoral neck [Colour figure can be viewed at wileyonlinelibrary.com]

1. *BMD at the lumbar spine*. Pooled analysis from seven trials (Han, 2019; Liu et al., 2019; Ma et al., 2014, 2018; Song et al., 2013; Wei, 2017; Zhang et al., 2018) reporting BMD values was statistically significant in favour of ZGP therapy plus anti‐osteoporosis drugs compared with anti‐osteoporosis drugs alone (WMD  =  0.09 g/cm^3^, 95% CI: 0.03 to 0.16, *p* = .006; heterogeneity: chi‐square (*χ* ^2^)  =  157.80, *p*  < .00001, *I* ^2^  =  96%). In addition, subgroup analysis for two trials (Liu et al., 2019; Wei, 2017) demonstrated that the effect of ZGP, combined with alendronate in improving BMD values at the lumbar spine, was superior to that of alendronate monotherapy (WMD  =  0.14 g/cm^3^, 95% CI: 0.04 to 0.25, *p*  = .006; heterogeneity: *χ* ^2^  =  18.06, *p*  < .00001, *I* ^2^  =  94%).

2. *BMD at the femoral neck*. The meta‐analysis of four trials (Han, 2019; Liu et al., 2019; Song et al., 2013; Wei, 2017) detected no significant difference in BMD values at the femoral neck under ZGP therapy plus anti‐osteoporosis drugs compared with anti‐osteoporosis drugs alone (WMD  =  0.08 g/cm^3^, 95% CI: −0.01 to 0.16, *p*  = .08; heterogeneity: *χ* ^2^  =  87.95, *p*  < .00001, *I* ^2^  =  97%). However, subgroup analysis for two trials (Liu et al., 2019; Wei, 2017) showed that the effect of ZGP, combined with alendronate, was not superior to that of alendronate monotherapy in improving the BMD values at the femoral neck (WMD  =  0.11 g/cm^3^, 95% CI: −0.01 to 0.23, *p*  = .07; heterogeneity: *χ* ^2^ = 31.74,  *p*  < .00001, *I* ^2^  =  97%). Figure 3 depicts the forest plot of ZGP plus alendronate compared with alendronate alone regarding BMD at the lumbar spine and femoral neck.

In addition, two other trials evaluated the effect of ZGP monotherapy and combination therapy on BMD values at the lumbar spine and femoral neck. One trial (Li et al., 2015) found no significant difference between the ZGP group and the alendronate group after a 2‐year follow‐up (*p* > .05). Nevertheless, one study did report statistical significance between ZGP and ZGP placebo groups on the basis of Caltrate tablets (*p* < .05) (Li & Zhang, 2018).

3. *BMD in Ward's area*. The meta‐analysis of three trials (Liu et al., 2019; Ma et al., 2018; Song et al., 2013) revealed significant effects of ZGP therapy plus anti‐osteoporosis drugs for improving BMD values in Ward's area compared with anti‐osteoporosis drugs alone (WMD  = 0.06 g/cm^3^, 95% CI: 0.02 to 0.10, *p*  = .002; heterogeneity: *χ* ^2^  =  8.15, *p* = .02, *I* ^2^  =  75%). However, only one study (Li & Zhang, 2018) reported that the effect of ZGP was better than that of placebo in slowing the decline in BMD in Ward's area on the basis of Caltrate tablets (*p* < .05).

4. *BMD at the total hip*. The combined effects of two independent trials (Ma et al., 2018; Wei, 2017) suggested that total hip BMD was significantly improved by ZGP compared with anti‐osteoporosis drugs (WMD  =  0.08 g/cm^3^, 95% CI: 0.05 to 0.11, *p*  < .00001; heterogeneity: *χ* ^2^  =  1.04, *p*  = .31, *I* ^2^  =  3%).

5. *BMD at the heel bone*. Two trials (Ma et al., 2018; Wang et al., 2014) evaluated BMD in the heel bone, but no meta‐analysis could be conducted. Moreover, no significant difference was found between ZGP and Caltrate tablets for diabetes‐induced osteoporosis (*p* > .05). However, a remarkable improvement in heel bone BMD with ZGP plus salmon calcitonin was identified compared to the use of salmon calcitonin alone (*p* < .05).

#### 3.4.3 Bone metabolism markers

Ten trials (Han, 2019; Li & Zhang, 2018; Liu et al., 2019; Ma et al., 2014, 2018; Peng, 2010; Wang et al., 2014; Wei, 2017; Yan et al., 2012; Zhang et al., 2018) evaluated bone metabolism markers.

**FIGURE 4:**
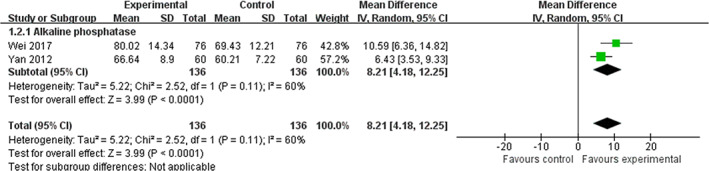
Forest plot of ZGP plus anti‐osteoporosis drugs compared with anti‐osteoporosis drugs alone with regard to ALP [Colour figure can be viewed at wileyonlinelibrary.com]

1. *Calcium and phosphorus metabolism indicators*. Calcium (Ca), phosphorus (P), parathyroid hormone (PTH), calcitonin (CT), and 25‐(OH)_2_D_3_ were mainly examined. A ZGP monotherapy study (Peng, 2010) indicated no significant difference in the level of blood Ca and P and the urine calcium and creatinine ratio. Four ZGP combination therapy studies demonstrated that ZGP plus conventional medicine could significantly improve the levels of urine Ca (Yan et al., 2012), CT (Ma et al., 2014), 1,25‐(OH)_2_D_3_ (Wei, 2017), and 25(OH)_2_D (Zhang et al., 2018) compared to conventional medicine, but differences in blood Ca and P (Ma et al., 2014) were not found between the groups.

2. *Bone formation markers*. Alkaline phosphatase (ALP), bone alkaline phosphatase (BALP), bone Gla protein (BGP), and procollagen type 1 N‐terminal propeptide (P1NP) were mainly assessed. A meta‐analysis of two trials (Yan et al., 2012; Wei, 2017) on combination therapy identified a significant ALP‐increasing effect compared with conventional medicine alone (WMD  =  8.21 μ/L, 95% CI: 4.18 to 12.25, *p*  < .0001; heterogeneity: *χ* ^2^  =  2.52, *p*  = .11, *I* ^2^  =  60%). Moreover, a meta‐analysis of three trials (Liu et al., 2019; Ma et al., 2014; Wei, 2017) revealed a significant lowering effect of combination therapy on BGP (WMD  =  −7.13 ng/ml, 95% CI:  −9.92 to −4.35, *p*  < .0001; heterogeneity: *χ* ^2^ =  91.36, *p*  < .0001, *I* ^2^  =  98%). Another meta‐analysis based on two trials (Liu et al., 2019; Ma et al., 2018) also found a significant lowering effect of combination therapy on BALP (WMD  =  −4.47 μ/L, 95% CI:  −6.23 to −2.72, *p* < .0001; heterogeneity: *χ* ^2^ =  2.12, *p*  = .15, *I* ^2^  =  53%). Figure 4 presents the forest plot of ZGP plus anti‐osteoporosis drugs compared with anti‐osteoporosis drugs alone with regard to ALP.

In one trial (Wang et al., 2014), bone biochemical markers were observed with ZGP treatment for diabetes‐induced osteoporosis. Based on hypoglycaemic therapy, ZGP was better than Caltrate tablets in improving the levels of PTH, ALP, and BGP (*p* < .05).

**FIGURE 5:**
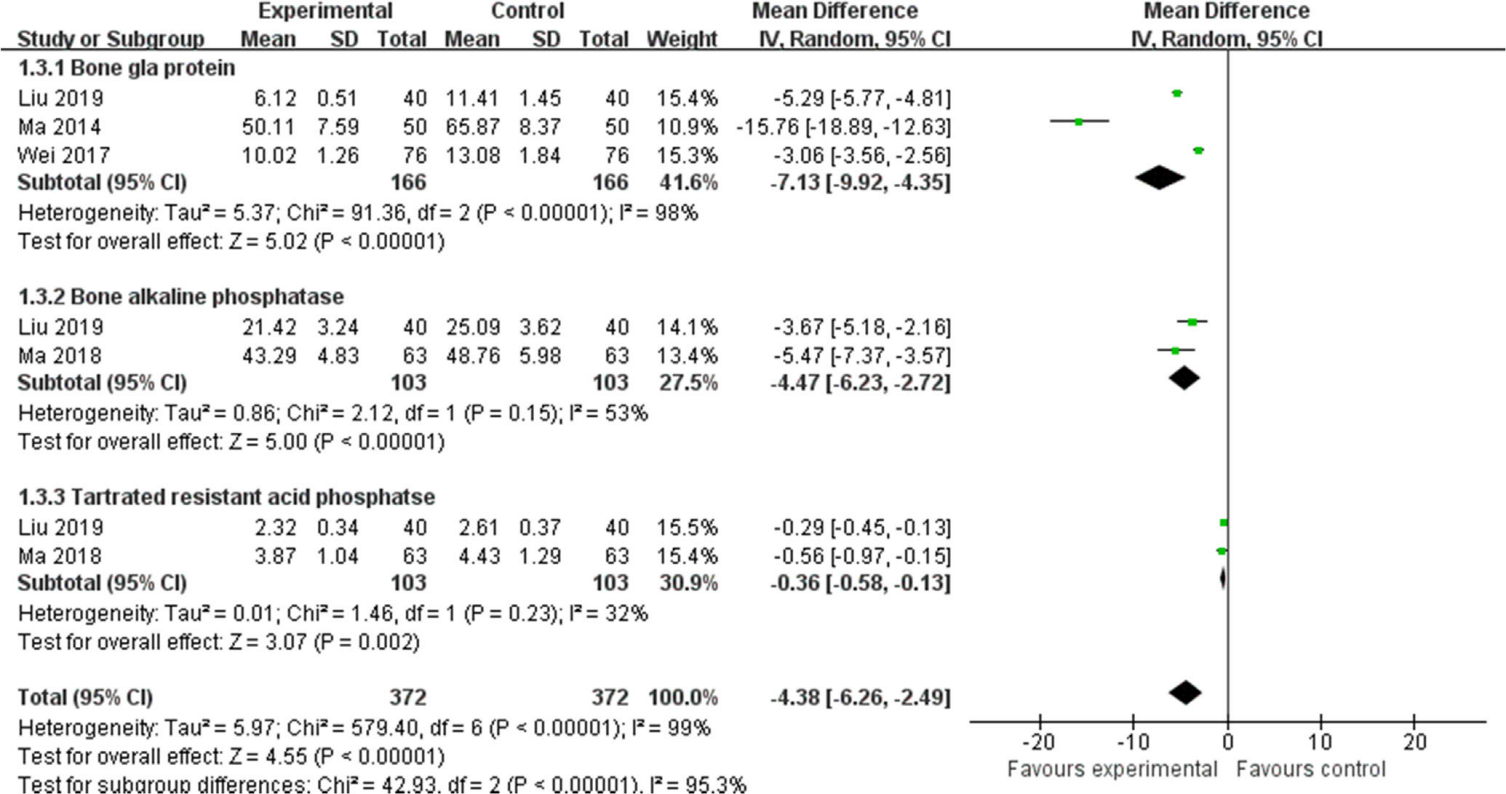
Forest plot of the effects of ZGP plus anti‐osteoporosis drugs compared with anti‐osteoporosis drugs alone for BGP, BALP, and TRACP [Colour figure can be viewed at wileyonlinelibrary.com]

3. *Bone resorption markers*. Tartrate‐resistant acid phosphatase (TRACP), TRACP‐5b, urine deoxypyridinoline and creatinine ratio (D‐Pyr/Cr), and β cross‐linked C‐telopeptide of type 1 collagen (β‐CTX) were mainly assessed. The pooled effect of two trials (Liu et al., 2019; Ma et al., 2018) showed a significant lowering effect of ZGP combined with anti‐osteoporosis drugs on TRACP compared to anti‐osteoporosis drugs alone (WMD  =  −0.36 μ/L, 95% CI:  −0.58 to −0.13, *p*  = .002; heterogeneity: *χ* ^2^  = 1.46, *p*  = .23, *I* ^2^  =  32%). Significant improvements in TRACP‐5b (Ma et al., 2018) and D‐Pyr/Cr (Han, 2019) were also found with combination therapy. Figure 5 presents the forest plot of the effects of ZGP plus anti‐osteoporosis drugs compared with anti‐osteoporosis drugs alone for BGP, BALP, and TRACP.

One study (Li & Zhang, 2018) further confirmed statistical significance in favour of ZGP compared to placebo on the basis of Caltrate tablets in improving P1NP, β‐CTX, and β‐catenin levels (*p* < .05).

4. *Hormones*. Only one trial (Liu et al., 2019) measured serum oestradiol (E2), follicle‐stimulating hormone (FSH), and luteinizing hormone (LH). A remarkable improvement in hormone levels with ZGP plus anti‐osteoporosis drugs was identified compared to anti‐osteoporosis drugs alone (*p* < .05).

5. *Cytokines*. A significant lowering effect on tumour necrosis factor α (TNF‐α) and cathepsin K (CTSK) was reported for combination therapy compared to anti‐osteoporosis agents alone (*p* < .05).

#### 3.4.4 Pain symptoms

Only one trial (Zhang et al., 2018) reported the results of the visual analogue scale (VAS) for assessing the degree of pain. The results showed that ZGP plus anti‐osteoporosis drugs were superior to anti‐osteoporosis drugs alone in alleviating pain symptoms (*p* < .05).

#### 3.4.5 Quality of life

The short form‐36 questionnaire (SF‐36), measuring quality of life, was reported in only one trial (Li & Zhang, 2018). A remarkable improvement in each domain with ZGP versus placebo was identified based on Caltrate tablets (*p* < .05).

#### 3.4.6 Adverse drug reaction

Six trials (Li et al., 2015; Ma et al., 2018; Peng, 2010; Wang et al., 2014; Wei, 2017; Zhang et al., 2018) reported the ADR of ZGP monotherapy and combination therapy. No adverse reactions occurred in two trials of ZGP for treating osteoporosis (Li et al., 2015; Peng, 2010). Three trials (Ma et al., 2018; Wei, 2017; Zhang et al., 2018) included the ADR of combination treatment, and one trial (Wang et al., 2014) reported no ADR in the combination group. ADRs of ZGP combined with anti‐osteoporosis drugs included gastrointestinal discomfort (nausea, diarrhoea), dizziness, and fatigue. The other six studies did not mention adverse events or ADRs.

### 3.5 Publication bias

The number of all enrolled studies was too small (less than 10) to assess publication bias for each outcome.

### 3.6 Evidence level

In our meta‐analysis, a high risk of methodological bias and suspected imprecision of the outcome, including BMD at all sites and bone metabolism markers, decreased the quality of the evidence. However, no upgraded factors were found. We graded the overall quality of available evidence through the GRADEpro Guideline Development Tool (GDT). The quality of evidence for all outcomes was graded as low, which was based on the rigorous evaluation for “Decreased quality of evidence” (risk of bias, inconsistency, indirectness, imprecision, and publication bias) and three items for “Increased quality of evidence” (large effect, plausible confounding would change the effect, and dose–response effect). Unfortunately, the risk of bias and imprecision for all studies were assessed as serious, mainly due to the limitations in study design, execution, and reporting. A summary of the strength of the evidence for the outcomes is presented in Table 4.

**TABLE 4:** The summary of the strength of evidence for outcomes

Certainty assessment							Summary of findings					
Participants (studies)	Risk of bias	Inconsistency	Indirectness	Imprecision	Publication bias	Overall certainty of evidence	Study event rates (%)		Relative effect (95% CI)	Anticipated absolute effects		
							With anti‐osteoporosis drugs alone	With ZGP plus anti‐osteoporosis drugs		Risk with anti‐osteoporosis drugs alone	Risk difference With ZGP plus Anti‐osteoporosis drugs	
**ZGP plus anti‐osteoporosis drugs compared to anti‐osteoporosis drugs alone for treating osteoporosis**												
BMD at all sites												
1,687 (7 RCTs)	Serious^a^	Not serious	Not serious	Serious^b^	None	⊕⊕◯◯ Low	842	845	—	The mean BMD at all sites was 0	MD 0.08 higher (0.05 higher to 0.12 higher)	
BMD at all sites—BMD at lumbar spine											
719 (7 RCTs)	Serious^a^	Not serious	Not serious	Serious^b^	None	⊕⊕◯◯ Low	359	360	—	The mean BMD at all sites—BMD at lumbar spine was 0	MD 0.09 higher (0.03 higher to 0.16 higher)	
BMD at all sites—BMD at femoral neck											
409 (4 RCTs)	Serious^a^	Not serious	Not serious	Serious^b^	None	⊕⊕◯◯ Low	204	205	**—**	The mean BMD at all sites—BMD at femoral neck was 0	MD 0.08 higher (0.01 lower to 0.16 higher)	
BMD at all sites—BMD at wards area											
281 (3 RCTs)	Serious^a^	Not serious	Not serious	Serious^b^	None	⊕⊕◯◯ Low	140	141	**—**	The mean BMD at all sites—BMD at wards area was 0	MD 0.06 higher (0.02 higher to 0.1 higher)	
BMD at all sites—BMD at total hip												
278 (2 RCTs)	Serious^a^	Not serious	Not serious	Serious^b^	None	⊕⊕◯◯ Low	139	139	**—**	The mean BMD at all sites—BMD at total hip was 0	MD 0.08 higher (0.05 higher to 0.11 higher)	
Bone metabolism markers(1)												
272 (2 RCTs)	Serious^a^	Not serious	Not serious	Serious^b^	None	⊕⊕◯◯ Low	136	136	**—**	The mean bone metabolism markers(1) was 0	MD 8.21 higher (4.18 higher to 12.25 higher)	
Bone metabolism markers(1)—alkaline phosphatase												
272 (2 RCTs)	Serious^a^	Not serious	Not serious	Serious^b^	None	⊕⊕◯◯ Low	136	136	**—**	The mean bone metabolism markers(1)—Alkaline phosphatase was 0	MD 8.21 higher (4.18 higher to 12.25 higher)	
Bone metabolism markers(2)												
744 (4 RCTs)	Serious^a^	Not serious	Not serious	Serious^b^	None	⊕⊕◯◯ Low	372	372	**—**	The mean bone metabolism markers(2) was 0	MD 4.38 lower (6.26 lower to 2.49 lower)	
Bone metabolism markers(2)—bone gla protein												
332 (3 RCTs)	Serious^a^	Not serious	Not serious	Serious^b^	None	⊕⊕◯◯ Low	166	166	**—**	The mean bone metabolism markers(2)—Bone Gla protein was 0	MD 7.13 lower (9.92 lower to 4.35 lower)	
Bone metabolism markers(2)—bone alkaline phosphatase												
206 (2 RCTs)	Serious^a^	Not serious	Not serious	Serious^b^	None	⊕⊕◯◯ Low	103	103	**—**	The mean bone metabolism markers(2)—Bone alkaline phosphatase was 0	MD 4.47 lower (6.23 lower to 2.72 lower)	
Bone metabolism markers(2)—Tartrated resistant acid phosphatase												
206 (2 RCTs)	Serious^a^	Not serious	Not serious	Serious^b^	None	⊕⊕◯◯ Low	103	103	**—**	The mean bone metabolism markers(2)—Tartrated resistant acid phosphatase was 0	MD 0.36 lower (0.58 lower to 0.13 lower)	
**ZGP plus alendronate compared to alendronate alone for treating osteoporosis**											
BMD at all sites											
464 (2 RCTs)	Serious^a^	Not serious	Not serious	Serious^b^	None	⊕⊕◯◯ Low	232	232	**—**	The mean BMD at all sites was 0	MD 0.13 higher (0.17 higher to 0.19 higher)
BMD at all sites—BMD at lumbar spine											
232 (2 RCTs)	Serious^a^	Not serious	Not serious	Serious^b^	None	⊕⊕◯◯ Low	116	116	**—**	The mean BMD at all sites—BMD at lumbar spine was 0	MD 0.14 higher (0.04 higher to 0.25 higher)
Bone metabolism markers(2)—Tartrated resistant acid phosphatase											
232 (2 RCTs)	Serious^a^	Not serious	Not serious	Serious^b^	None	⊕⊕◯◯ Low	116	116	**—**	The mean BMD at all sites—BMD at femoral neck was 0	MD 0.11 higher (0.01 lower to 0.23 higher)

Abbreviations: CI, confidence interval; MD, mean difference.

^a^ All studies were assessed to be high risk of bias.

^b^ In the meta‐analysis, the suspected imprecision for the outcome including BMD at all sites and bone metabolism markers decreased the quality of evidence.

## 4 DISCUSSION

As the most common skeletal disease, the prevalence of osteoporosis is gradually increasing with the ageing of the global population (Xia et al., 2019). Severe osteoporosis often leads to osteoporotic fractures (Lu, Ren, Liu, Xu, & Liu, 2019). As clinical practice and research develop, Chinese herbal formulas have attracted increasing attention for the treatment of osteoporosis (He, Chen, & Lin, 2017; Shi et al., 2017; Zhu et al., 2012). Among classic herbal formulas, ZGP is among the most typical formulas that mainly treat osteoporosis with kidney‐yin deficiency syndrome according to the theory of TCM (Li et al., 2018). The results of the current meta‐analysis based on available evidence from 12 articles showed that ZGP combined with anti‐osteoporosis drugs significantly increased BMD at the lumbar spine, Ward's area, and total hip. By improving bone metabolism markers, ZGP plus anti‐osteoporosis drugs also dramatically improved the levels of alkaline phosphatase, BGP, BALP, and TRACP. Nonetheless, an exact and uniform conclusion for the other outcomes cannot be drawn from existing information. In the safety evaluation of herbal formulas, ZGP combined with anti‐osteoporosis drugs might produce fewer and mild gastrointestinal discomfort. ADRs of ZGP used independently were not observed in two trials, with no severe adverse reactions. Regardless, according to the GRADE evaluation, the overall quality of evidence for the meta‐analysis was low because of the limitations of the study methods and imprecision in the studies.

For osteoporosis, the incidence of fracture should be the endpoint outcome and most important evaluation index (Liu et al., 2014). In our meta‐analyses, the primary outcome was fracture incidence. However, the primary outcome in a newly published study protocol of a systematic review by Chen et al. (Chen et al., 2019) was BMD. Nevertheless, more detailed indicators and subgroup analyses are provided in our review. Indeed, BMD at different sites, a variety of bone metabolism markers, clinical symptoms, quality of life, and adverse events or ADR were fully considered in this systematic review. Furthermore, subgroup analysis demonstrated that ZGP plus alendronate was superior to alendronate alone in improving BMD values at the lumbar spine, although there was no difference in BMD values at the femoral neck.

However, some limitations of this study should be noted. First, as the sample size of the included studies involved fewer than 200 cases, the cumulative population was small, which was not sufficient to provide high‐quality evidence to confirm the clinical effect of ZGP monotherapy and combination therapy for osteoporosis. Second, although we conducted a rigorous assessment of the quality of the included literature, significant statistical heterogeneity still existed among the meta‐analysis of the combination treatment for BMD at different sites and bone metabolism markers. In addition, some potential bias inherent in the original clinical research, such as the clinical diversity among trials (the type of osteoporosis and anti‐osteoporosis drugs), may be present. Moreover, the majority of the studies enrolled had methodological limitations, including single‐centre RCTs without placebo‐controlled evidence. At the same time, we did not interview the authors of the studies by telephone or email for more detailed information. Third, all studies were found in the Chinese literature because ZGP studies have mainly been conducted in mainland China. This may cause a certain bias regarding the results of this study. In short, more RCTs with large samples and of high quality are needed to verify the results of this meta‐analysis.

## 5 CONCLUSION

Our pooled results show that ZGP combined with anti‐osteoporosis drugs may have beneficial effects on osteoporosis with respect to BMD and bone metabolism markers. There were no ADRs when using ZGP alone, but ADRs of ZGP combined with anti‐osteoporosis drugs included gastrointestinal discomfort (nausea, diarrhoea), dizziness, and fatigue. However, a definite conclusion regarding other indicators cannot be drawn from the existing information. The results of this review demonstrate that ZGP is a potential candidate for osteoporosis treatment, although the quality of evidence remains weak.

## CONFLICT OF INTEREST

The authors have no conflicts of interest.

## Supporting information


**Data S1**: Supporting information.
