# Supplementary material for: An evaluation of the effects and safety of Zuogui pill for treating osteoporosis: Current evidence for an ancient Chinese herbal formula
Source: Phytother Res. 2020 Oct 21;35(4):1754–67. doi: 10.1002/ptr.6908 (PMC8246738; doi:10.1002/ptr.6908)
Supplement: Supplementary file 1 — Data S1: Supporting information. [file PTR-35-1754-s001.docx]

**Supplementary Material**

Search Strategy for English and Chinese databases(Take PubMed database and China National Knowledge Infrastructure database as examples)

**1.The detailed search terms in PubMed were as follows:**

#1. (Zuogui Pill[Title/Abstract]) OR (Zuogui granules[Title/Abstract])

#2. ((((((((((osteoporosis [MeSH Terms]) OR (osteoporosis [Title/Abstract])) OR (primary osteoporosis [Title/Abstract])) OR (postmenopausal osteoporosis [MeSH Terms])) OR (postmenopausal osteoporosis [Title/Abstract])) OR (senile osteoporosis [Title/Abstract])) OR (age-related osteoporosis [Title/Abstract])) OR (bone loss [Title/Abstract])) OR (osteopenia [Title/Abstract])

#3. ((((randomized [Text Word] OR random [Text Word]) OR controlled [Text Word]) OR control [Text Word]) OR trial [Text Word]

#4. #1AND#2AND#3 Filters: Clinical trial; Humans.

Limits: Jan. 2020

**2.** **中文数据库中国知网的检索策略如下(The detailed search terms in China National Knowledge Infrastructure database were as follows)**

(((主题= (骨质疏松+绝经后骨质疏松症+老年性骨质疏松症) 或者 题名= (骨质疏松+绝经后骨质疏松症+老年性骨质疏松症) 或者 v_subject= (中英文扩展(骨质疏松)+中英文扩展(绝经后骨质疏松症)+中英文扩展(老年性骨质疏松症)) 或者 title= (中英文扩展(骨质疏松)+中英文扩展(绝经后骨质疏松症)+中英文扩展(老年性骨质疏松症))) 并且 (主题= (左归丸+左归饮+左归片) 或者 题名= (左归丸+左归饮+左归片) 或者 v_subject= (中英文扩展(左归丸)+中英文扩展(左归饮)+中英文扩展(左归片)) 或者 title= (中英文扩展(左归丸)+中英文扩展(左归饮)+中英文扩展(左归片)))) 并且 (主题= (随机+临床+对照) 或者 题名= (随机+临床+对照) 或者 v_subject= (中英文扩展(随机)+中英文扩展(临床)+中英文扩展(对照)) 或者 title= (中英文扩展(随机)+中英文扩展(临床)+中英文扩展(对照)))) (模糊匹配)

检索截止日期：2020年1月31日
